# Treatment of Boerhaave syndrome: experience from a tertiary center

**DOI:** 10.1007/s00464-025-11540-8

**Published:** 2025-02-10

**Authors:** Cezanne D. Kooij, Eleni Boptsi, Bas L. A. M. Weusten, D. R. de Vries, Jelle P. Ruurda, Richard van Hillegersberg

**Affiliations:** 1 Department of Surgery, University Medical Center Utrecht, Utrecht, The Netherlands; 2 Department of Gastroenterology, University Medical Center Utrecht, Utrecht, The Netherlands; 3 Heidelberglaan 100, 3584 CX Utrecht, The Netherlands

**Keywords:** Boerhaave syndrome, Spontaneous esophageal perforation, Esophageal perforation

## Abstract

**Background:**

Boerhaave syndrome is a rare, life-threatening condition, characterized by spontaneous esophageal rupture. This study aims to share our 13-year experience in managing Boerhaave syndrome.

**Methods:**

A retrospective, observational study was conducted of consecutive patients with Boerhaave syndrome who presented at our tertiary referral center, between 2011 and 2023. Patients were categorized by time to diagnosis, to assess the impact of diagnostic delay.

**Results:**

Among 21 patients, 13 (62%) were diagnosed early (< 24 h) and 8 (38%) late (> 24 h). In the early-diagnosed group (*n* = 13), 6 patients (46%) received primary intervention with stent placement in combination with surgical drainage (5 with mediastinal and pleural drainage and 1 with only pleural drainage), while 5 patients (38%) were initially treated with only a stent. One patient (8%) underwent surgical pleural drainage alone and one (8%) underwent an esophagectomy. Among the 8 late-diagnosed patients, 4 (50%) were primarily treated with both stent placement and surgical drainage (2 with mediastinal drainage, 1 with pleural drainage and 1 with both), 3 (38%) with only stent placement, and one (13%) was managed conservatively. Additional interventions were required in 14 patients (67%). Additional surgical drainage was performed in 5 of 8 patients who had initially been treated with stent only (63%) and in 2 of 10 patients who had initially received both stent and surgical drainage (20%). Stent complications occurred in 7 patients (37%), including leakage (16%), migration (16%), and bleeding (5%). The median hospital stay was 32 days (IQR 15–37) and the overall 90-day mortality was 14%. Mortality was significantly higher in late-diagnosed patients (*n* = 3, 38%) compared to those early diagnosed (*n* = 0, 0%) (*p* = 0.042), with all 3 deceased patients either refusing or being unfit for treatment.

**Conclusion:**

Based on this study, we recommend prioritizing closure of the defect combined with drainage, while considering individual patient factors, including advanced age.

The most common cause of esophageal perforation is iatrogenic, accounting for up to 70% of esophageal perforation, followed by trauma, spontaneous, and foreign body [1]. Boerhaave syndrome, a rare yet life-threatening medical emergency, is characterized as a spontaneous esophageal perforation. It is typically associated with severe straining or vomiting, where a sudden increase in intraesophageal pressure, combined with negative intrathoracic pressure (as in normal physiological state), leads to esophageal rupture [2]. Boerhaave syndrome represents about 15% of esophageal perforations [3]. Patients often present with non-specific symptoms, leading to a high incidence of initial misdiagnosis [4]. Typically, the perforation leads to mediastinitis due to the contamination of the mediastinal cavity from gastric contents and food, thereby leading to sepsis and potential multiple organ failure and a high mortality rate that varies from 11% up to 50% [2, 5–8]. The primary objectives in the management of Boerhaave syndrome include prompt sealing of the perforation, mediastinal and pleural or abdominal drainage combined with antibiotics to control the sepsis, and nutritional support [9].

Treatment decisions should be based on the patient’s clinical condition upon presentation, current comorbidities, and time of diagnosis. Primary surgical repair, with or without the use of reinforcement flaps, is considered the standard procedure, but it is mainly limited to patients presenting within the first 24 h since the onset of symptoms [10]. Other options include surgical drainage, with a preference for minimally invasive techniques. Ultimately, esophageal resection can be considered. Non-surgical strategies vary from conservative management with antibiotics to endoscopically placed stents or clips. The use of self-expanding metal stents (SEMS) is well established in the palliative treatment of malignant esophageal stenosis and has emerged as an alternative treatment option for benign esophageal strictures [11, 12]. In addition, many studies have demonstrated the efficacy of partially or fully covered SEMS, in managing upper gastrointestinal leaks [11, 13–16]. This technique was therefore increasingly used over the past years in our center.

Due to the rarity of Boerhaave syndrome, there are currently no definitive management guidelines. The objective of this study is to share the experience of a tertiary (referral) hospital in managing patients with Boerhaave syndrome over a span of 13 years and to provide clinical guidance.

## Methods

A single-center retrospective, observational study was conducted that included consecutive patients with Boerhaave syndrome who presented in the University Medical Center Utrecht (Utrecht, The Netherlands) from January 2011 to December 2023. Boerhaave syndrome was defined as a spontaneous perforation/rupture of the esophagus without previous iatrogenic intervention, surgery, and/or trauma. Patients with esophageal perforations due to causes other than Boerhaave syndrome such as malignancy or caustic injury were excluded. Patients were identified through free text searching using Emerse, an electronic patient records search engine designed for analyzing free text data [17]. All information on patient characteristics and outcomes was obtained from the HiX electronic patient record system (version 6.3; ChipSoft B.V., Amsterdam, The Netherlands). Keyword terms “Boerhaave” and “Boerhave” were utilized to ensure comprehensive identification of relevant cases. The ethical board approved this study allowing patients to be included unless they objected (document number 23U-0578). This manuscript was composed using the STROBE checklist [18].

### Outcomes and data collection

The primary outcome of this study was the initial treatment modality in relation to the time of diagnosis. Secondary outcomes included the 30-day and 90-day mortality rate, length of hospital stay, duration of ICU stay, and the incidence of complications.

Baseline characteristics, including age at the time of treatment, gender, BMI, and comorbidities, were gathered. Additionally, information regarding initial diagnosis, diagnostic methods, delay in diagnosis, clinical presentation, characteristics of the perforation, treatment provided, mortality, and outcomes were collected. To assess the impact of diagnostic delay, patients were categorized into two groups based on the time of diagnosis: early diagnosis (within 24 h of symptom onset) and late diagnosis (after 24 h). The duration of hospital stay, Intensive Care Unit (ICU) stay, required additional interventions, morbidity (according to Clavien–Dindo classification), and mortality were taken into account as treatment outcomes. Long-term complications were also documented up to the last follow-up. Esophageal stenosis was defined as dysphagia requiring dilatation at the site of prior intervention [19].

The Pittsburgh perforation severity score (PSS), designed to measure the severity of esophageal disruption and to aid in management decisions of esophageal perforation, was retrospectively calculated from data and findings at the time of presentation. Each component contributes to a maximum score of 18 points as following: one point was assigned for age > 75 years, tachycardia (> 100 bpm), leukocytosis (> 10.000 white blood cells/mL) or pleural effusion (on chest X-ray, computed tomography, or barium swallow); two points for fever (> 38.5 °C), non-contained leak (mediastinitis and/or mediastinal/pleural fluid collections on computed tomography), respiratory compromise (respiratory rate > 30, increasing oxygen requirement, or need for mechanical ventilation), or time to diagnosis > 24 h from symptoms onset; and three points for the presence of cancer or hypotension (SBP < 90 mmHg) [20]. Primary intervention was defined as the procedure(s) aimed at sealing the perforation and/or drainage of contaminated collections and an additional intervention was defined as any subsequent procedure(s) performed to achieve similar goals. Re-stenting, when used as part of the stenting treatment to assist in removing the initial stent or to address a persistent defect, was not considered an additional intervention since it is part of the initial treatment strategy. However, if (re-)stenting was performed for a different reason, it was considered an additional intervention. Treatment success was defined as the absence of esophageal leakage following treatment and the patient being discharged alive.

### Statistical analysis

All statistical analyses were performed using the SPSS version 29.0 statistical software package (IBM Corp., New York, USA). Continuous variables were reported as the mean ± standard deviation (SD) or median (IQR), as appropriate. Categorical variables were expressed as *n* (%). Univariable analysis was performed with Fisher’s exact test for categorical variables and independent *t* test or Mann–Whitney *U* test for continuous variables according to data distribution. A two-sided *p* value < 0.05 was considered statistically significant.

## Results

A total of 55 patients were identified through free text searching of the electronic patient files. The screening through radiology reports, multidisciplinary team meetings, and endoscopy reports yielded 26 patients who were reported to be diagnosed with Boerhaave syndrome upon their admission to the University Medical Center Utrecht and their medical records were reviewed. One patient was ultimately diagnosed with a mediastinal abscess from complicated diverticulitis and was therefore excluded from the cohort. Another patient experienced esophageal perforation following hematemesis, which was determined to be due to a gastric adenocarcinoma. A third patient, who did not have endoscopically confirmed perforation but rather a laceration, was also excluded. Additionally, two patients diagnosed with acute esophageal necrosis (black esophagus) were excluded.

Finally, 21 patients with Boerhaave syndrome were included in the cohort. A total of 4 patients (19%) were directly admitted to our center. Of the remaining 17 (81%) patients who were referred from another hospital, 2 (10%) patients were initially treated in the referring center. The mean age of the patients was 63 (± 18.3) years and 12 (57%) of them were male. In total, 19 (90%) patients had at least one comorbidity, among them 12 (57%) patients had cardiovascular disease and there were 3 (14%) patients with a concomitant esophageal disorder, namely eosinophilic esophagitis, Barrett esophagus, and previous Boerhaave syndrome. Additionally, 6 (29%) patients had a history of chronic alcohol consumption. Vomiting in 13 (62%) and chest pain in 12 (57%) patients were the most common presenting symptoms. A total of 4 (19%) patients presented with hemodynamic instability treated with fluids or subsequently with inotropic support, while 3 (14%) patients developed respiratory insufficiency necessitating intubation. In the majority of patients (19, 91%), the esophageal defects were located in the distal esophagus, and the median defect length was 3.5 cm (IQR 2.4–5.8). Table 1 demonstrates the baseline characteristics and details on presentation and diagnostics of the study population. A thoracic CT scan was performed in all patients. Also, all patients underwent upper gastrointestinal endoscopy to confirm the perforation and assess the feasibility of stent placement. Within this cohort, 13 (62%) patients received an early diagnosis and 8 (38%) patients a late diagnosis. Moreover, 7 (33%) cases were initially misdiagnosed, and subsequently admitted, under other medical specialties and/or received alternative treatments. The diagnoses assigned are outlined in Table 2. After retrospectively calculating the PSS score, we identified 1 (5%) patient with low score, 6 (29%) with medium scores and 11 (52%) with high score. No clear correlation between the PSS score and treatment outcomes could be identified. While patients treated operatively generally had higher scores, one patient with a score of 3 was treated conservatively without any adverse events and a patient with a low score (PSS 2) underwent VATS and stenting.

**Table 1:** Clinicopathologic characteristics of patients with Boerhaave syndrome (*n* = 21)

Age, y (mean [SD])	63 (± 18.3)
Gender, *n* (%)	
Male	12 (57)
Female	9 (43)
BMI, kg/m^2^ (mean [SD])	25.1 (± 4.4)
Chronic alcohol consumption, *n* (%)	6 (29)
Any comorbidity, *n* (%)	19 (90)
Pulmonary comorbidity	3 (14)
Cardiovascular comorbidity	12 (57)
Thromboembolic comorbidity	7 (33)
Gastrointestinal comorbidity	9 (43)
Endocrinological comorbidity (incl. DM)	5 (24)
Urological comorbidity	5 (24)
Neurological comorbidity	6 (29)
Concomitant esophageal disorder, n (%)	3 (14)
Previous Boerhaave syndrome	1 (5)
Eosinophilic esophagitis	1 (5)
Esophagus Barrett	1 (5)
Presentation, *n* (%)	
Vomiting	13 (62)
Chest pain	12 (57)
Abdominal pain	10 (48)
Dyspnea	9 (43)
Hematemesis	3 (14)
Pain radiating to the back	8 (38)
Subcutaneous emphysema	2 (10)
Time to diagnosis	
Early diagnosis (< 24 h)	13 (62)
Late diagnosis (> 24 h)	8 (38)
Side of perforation, *n* (%)	
Left	9 (43)
Right	7 (33)
Unclear	5 (24)
Perforation location, *n* (%)	
Proximal	1 (5)
Mid	1 (5)
Distal	19 (91)
Size of perforation in cm (median [IQR])	3.5 (2.4–5.8)
PSS, *n* (%)	
Low (0–2)	1 (5)
Medium (3–5)	6 (29)
High (> 5)	11 (52)
Unknown	3 (14)

*SD* standard deviation, *IQR* interquartile range, *PSS* Pittsburgh perforation severity score

**Table 2:** Initial diagnostic evaluations of Boerhaave syndrome patients

Initially misdiagnosed cases, *n* (%)	7 (33)
Alternative diagnoses	
Pulmonary embolism	1 (5)
Pneumonia	2 (10)
Esophageal tumor	1 (5)
Kidney stones	1 (5)
Non-specified abdominal pain	1 (5)
Dehydration after multiple vomiting	1 (5)

### Treatment strategies

Prior to the initial intervention, all patients were managed with intravenous antibiotics, fluids, and nil per mouth. In 8 patients (38%), a thoracic drain was placed before referral to our center. Nutritional support was provided to 18 patients (86%) via enteral or parenteral feeding. Enteral support was administered with feeding jejunostomy or duodenal tubes. Patients with mediastinitis were treated, with antibiotics for 6 weeks. Following treatment and initial recovery, 6 (27%) patients were transferred back to their local hospital for the remaining recovery period.

**Fig. 1:**
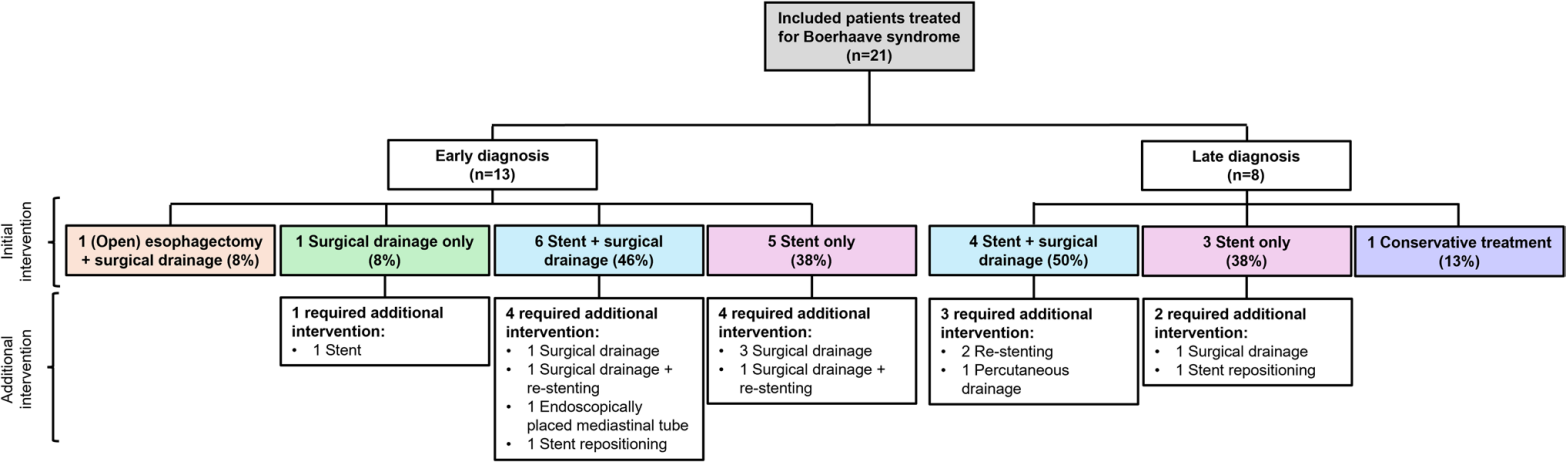
Flowchart of the initial treatment strategies and performed additional interventions of the included patients with Boerhaave syndrome (*n* = 21), subdivided into early- (*n* = 13) and late-diagnosed patients (*n* = 8)

Figure 1 outlines the primary treatment modalities, shown separately for early- and late-diagnosed patients, along with the required additional interventions.

In the early (< 24 h)-diagnosed patients (*n* = 13), endoscopic treatment with placement of a stent combined with surgical drainage was the primary intervention in 6 patients (46%). Surgical mediastinal and pleural drainage was performed in 4 patients via video-assisted thoracoscopic surgery (VATS). In the other 2 patients mediastinal and pleural, as well as solely pleural drainage, were performed via laparoscopic transhiatal approach. Surgical closure deemed feasible in one of these patients, where the perforation site was identified and sutured over the stent. Additional surgical drainage was required in 2 patients due to mediastinal abscess and pleural empyema, with one requiring re-stenting due to leakage caused by a lack of full coverage. Endoscopic repositioning of the stent was necessary in another patient due to migration. Also, a persistent mediastinal collection was drained with an endoscopically placed tube in one case. Of the other early-diagnosed patients, 5 (38%) were treated with a stent only; 4 of these required additional surgical drainage for pleural empyema or mediastinal abscess. Due to leakage, one of these 4 patients required a re-stenting procedure. One patient (5%) was initially treated with transhiatal pleural surgical drainage only but later required stenting. The remaining patient (5%) was treated with esophagectomy due to a large perforation extending from a partially ischemic distal esophagus to proximal stomach, thus identified as not suitable for stent treatment during endoscopy. Reconstruction by colonic interposition was performed three months later.

Of the late (> 24 h)-diagnosed patients (*n* = 8), 4 patients (50%) were also primarily treated endoscopically with placement of a stent in combination with surgical drainage. For surgical drainage, 3 patients underwent VATS: one had mediastinal drainage, another had pleural drainage, and the third had both. Additionally, one patient had mediastinal drainage performed via a laparoscopic transhiatal approach. Among these, 2 required re-stenting due to stent leakage and migration. Percutaneous drainage was also implemented in one patient in this treatment group for extrathoracic drainage. Initial treatment with only a stent was conducted in 3 patients (38%); one of these required surgical drainage due to pleural empyema and another required endoscopic repositioning following stent migration. One (5%) patient was successfully treated conservatively with antibiotics due to the small size of the tear and good clinical condition, with an uneventful recovery.

**Fig. 2:**
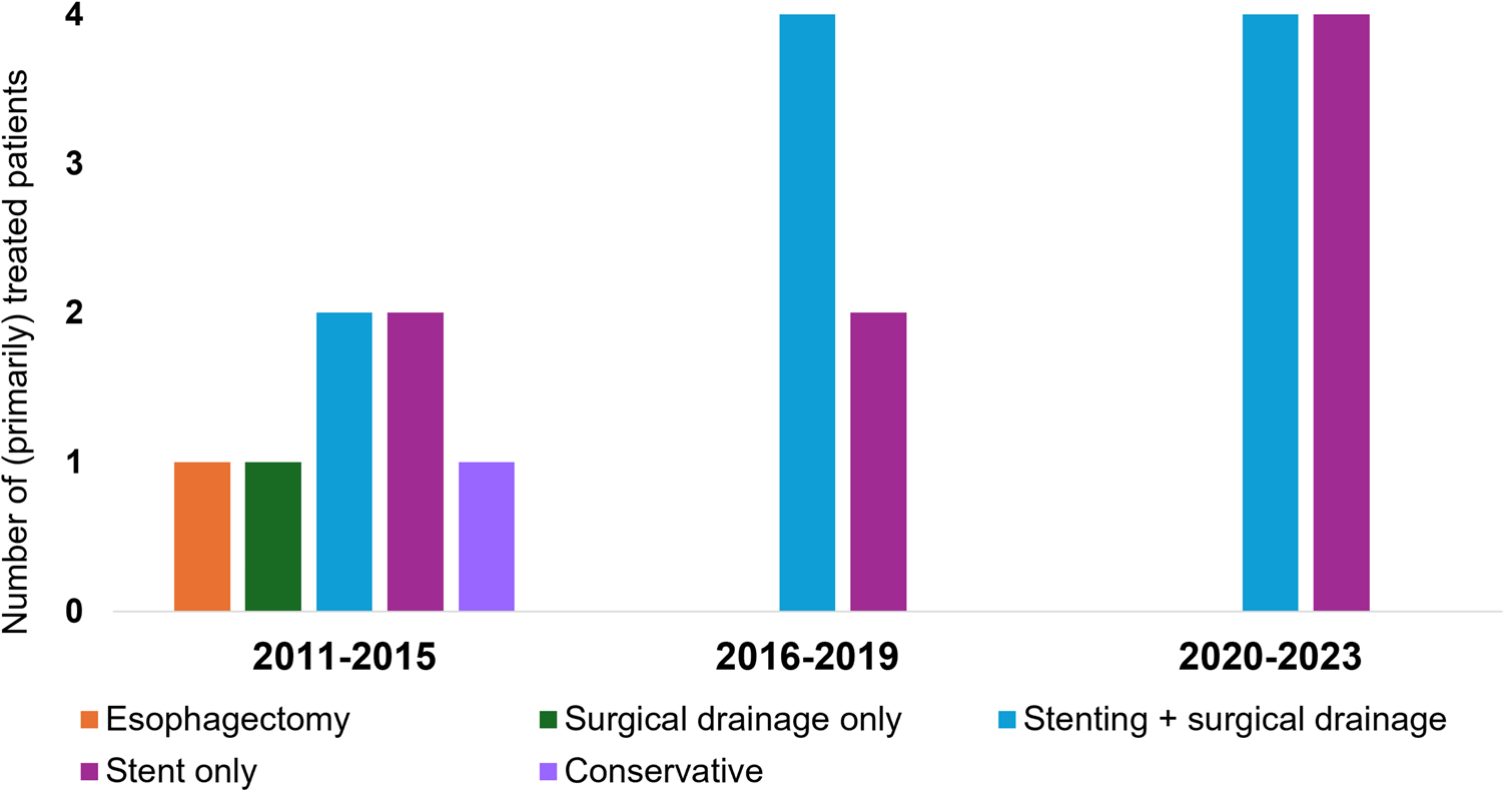
Overview per time period (2011–2015, 2016–2019, and 2020–2023) of initial treatment strategies in patients with Boerhaave syndrome (*n* = 21)

Figure 2 illustrates the initial treatment strategies over time, divided into three time frames, showing that in the most recent periods, the preferred treatment strategies were stenting combined with surgical drainage or stent placement only.

### Post-intervention outcomes

The treatment success rate in our cohort was 86% (18 out of 21 patients). The median length of hospital stay for patients with early diagnosis was 36 days (IQR 25–40), in comparison to 28 days (IQR 12–36) in patients diagnosed late (*p* = 0.317). Of the 13 patients diagnosed early, 8 (62%) were admitted in the ICU and the median length of ICU stay was 7 (IQR 4–10) days. Of the late-diagnosed patients, ICU stay was necessary in 5 (63%) of the cases and the median length of ICU stay was 6 days (IQR 4–12) (*p* = 0.293).

In total, 19 (90%) patients were treated with at least 1 stent, all using partially covered self-expandable metallic stents to seal the perforation. The median number of stents placed per patient was 2 (IQR 1–3) with a median duration until stent removal of 31 (IQR 24–45) days. Stent complications occurred in 7 of these 19 patients (37%). In total, 3 patients (16%) experienced leakage due to damaged stent covering and 3 (16%) stent migration, 2 of which required endoscopic repositioning. One patient experienced leakage twice. Bleeding occurred in one patient during the endoscopy for stent removal. The patient was admitted for observation, but no further intervention was needed. Stent complications are presented in Table 3.

**Table 3:** Details of stent treatment and associated complications in patients treated with at least one stent (*n* = 19)

Removal of the stents in days (median [IQR])	31 (24–45)
Stents placed (median [IQR])	2 (1–3)
Size of stent*	
23 × 120 mm	17 (89)
18 × 120 mm	1 (5)
23 × 155 cm	1 (5)
Patients with stent complications, n (%)	7 (37)
Leakage due to damaged stent covering	3 (16)
Stent migration	3 (16)
Bleeding	1 (5)
Interventions due to stent complications	7
Re-stenting	5
Repositioning	2

*IQR* interquartile range

*Size of the first placed stent (total; *n* = 19)

Only one patient was readmitted within 30 days of discharge for a stent removal. Due to stent ingrowth, a stent-in-stent procedure was performed. This case was complicated further by pneumonia, which was successfully treated with antibiotics, and the patient could ultimately be discharged in good condition (see Table 4).

**Table 4:** Post-intervention outcomes and follow-up of Boerhaave patients (*n* = 21)

Post-intervention stay	
Hospital stay, in days (median [IQR])	32 [15–37]
ICU stay, in days (median [IQR])^*^	6 [4–10]
Additional intervention, *n* (%)	14 (67)
Complications, *n* (%)^^^	18 (86)
Esophageal leakage	4 (19)
Abscess	6 (29)
Empyema	5 (24)
Pneumonia	5 (24)
ARDS	1 (5)
Atrial arrhythmia	4 (19)
Pulmonary embolism	2 (10)
Intestinal Ischemia	1 (5)
Esophagopleural fistula	1 (5)
Clavien–Dindo classification, *n* (%)	
I	0 (0)
II	0 (0)
IIIa	4 (19)
IIIb	8 (38)
IVa	4 (19)
IVb	0 (0)
V	2 (10)
In hospital mortality, *n* (%)	1 (5)
Readmission within 30 days, *n* (%)	1 (5)
30-day mortality, *n* (%)	2 (10)
90-day mortality, *n* (%)	3 (14)
Follow-up, in months (median [IQR])	3 (2–10)

^*^ICU admission was required in 13 (62%) patients

^^^Patients with one or more complications

The characteristics and outcomes of patients according to early or late diagnosis are presented in Table 5. No statistically significant differences were observed in age, gender, comorbidities, perforation size, or location between the two groups. The length of hospital stay, ICU stay, and the complication rates also did not differ significantly.

**Table 5:** Characteristics and outcomes according to diagnosis delay in Boerhaave patients (*n* = 21)

	Early diagnosis (< 24 h) *N* = 13	Late diagnosis (> 24 h) *N* = 8	*p* value
Age, y (mean [SD])	60.5 (14.9)	66.9 (23.5)	0.456^t^
Gender, *n* (%)			0.673^a^
Female	8 (62)	4 (50)	
Male	5 (38)	4 (50)	
BMI, kg/m^2^ (median [IQR])	25.5 (21.9–29.9)	23.2 (20.7–26.2)	0.272^b^
Chronic alcohol consumption, *n* (%)	4 (31)	2 (25)	1.000^a^
Any comorbidity, *n* (%)	12 (92)	7 (88)	1.000^a^
Cardiovascular disease	4 (31)	6 (75)	0.080^a^
Thromboembolic disease	4 (31)	3 (38)	1.000^a^
Perforation location, *n* (%)			1.000^a^
Proximal	1 (8)	0 (0)	
Mid	1 (8)	0 (0)	
Distal	11 (85)	8 (100)	
Size of perforation in cm (median [IQR])	3 (2.5–5)	4 (2–10)	0.855^b^
Initial intervention, *n* (%)			0.858^a^
Esophagectomy and surgical drainage	1 (8)	0 (0)	
Stent and surgical drainage	6 (46)	4 (50)	
Surgical drainage only	1 (8)	0 (0)	
Stent only	5 (38)	3 (38)	
Conservative	0 (0)	1 (13)	
Hospital stay, in days (median [IQR])	36 (25–40)	28 (12–36)	0.317^b^
ICU stay, in days (median [IQR])	7 (4–10)	6 (4–12)	0.293^b^
Complications, *n* (%)	11 (85)	7 (88)	1.000^a^
Esophageal leak	2 (15)	2 (25)	0.618^a^
Abscess	4 (31)	2 (25)	1.000^a^
Empyema	3 (23)	2 (25)	1.000^a^
Disease-specific mortality, *n* (%)	0 (0)	3 (38)	0.042^a^

^t^Independent samples *T* test

^a^Fisher’s exact test

^b^Mann–Whitney *U* test

### Mortality and follow-up

Patients were on regular follow-up, typically for a median of 3 months. During follow-up, two patients developed reflux esophagitis following multiple stent placement, treated with proton pump inhibitors (PPIs). One patient exhibited distal esophageal stenosis on swallow evaluation but required no further intervention. The patient resumed oral intake with no complaints.

Mortality due to Boerhaave syndrome in patients with late diagnosis was significantly higher (*n* = 3, 38%) compared to those diagnosed early (*n* = 0, 0%) (*p* = 0.042). This higher mortality appears to be primarily associated with patients declining further treatment rather than solely (the late diagnosis of) the condition itself. One patient, aged 94 years, declined further interventions after receiving a stent and opted for palliative treatment, passing away shortly after transfer to hospice care. In a 93-year-old patient, surgical intervention was deemed inappropriate given his advanced age and poor prognosis resulting from a late diagnosis of more than 72 h. Despite stenting and conservative management with thoracic drainage, the patient died after 13 days of hospital stay due to multi-organ failure. The third patient, aged 77, experienced stent dislocation leading to gastrointestinal bleeding after re-stenting due to a persistent defect. The patient refused further interventions and died of hypovolemic shock.

## Discussion

Boerhaave syndrome remains a life-threatening emergency that requires early recognition and aggressive treatment. Its rarity hinders the development of a standardized treatment algorithm. Recent trends in treatment have shifted toward less invasive techniques, including minimally invasive surgery and endoscopic interventions. Recent literature suggests a treatment strategy of source control that combines closure, either surgically or endoscopically, with drainage [8, 16, 21–23]. In our cohort, early stenting combined with minimally invasive surgical drainage was the most frequently performed treatment strategy, which resulted in favorable outcomes. Additionally, our study indicates that timely diagnosis is a critical factor in reducing mortality among these patients. However, it is important to note that the patients who died in our cohort were primarily of advanced age and/or refused further treatment.

Misdiagnosis is a main concern in the management of Boerhaave syndrome [16]. The typical symptoms include vomiting, chest pain, and subcutaneous emphysema. However, these symptoms occur in only 14% of cases according to the literature [24]. In our study, we reported a high frequency of vomiting and chest pain, followed by less specific symptoms as abdominal pain and dyspnea. Additionally, 33% of our cases were initially misdiagnosed, with pulmonary disorders being the most common alternative diagnosis. Physicians need to be cautious when patients present with specific (vomiting, chest pain, subcutaneous emphysema), but also with non-specific symptoms (dyspnea, abdominal pain), always considering Boerhaave syndrome in the differential diagnosis, particularly among older patients with a history of alcohol abuse [6, 25].

Time from symptom onset to diagnosis is a major prognostic factor in mortality and morbidity of Boerhaave syndrome [14]. Late diagnosis is associated with a significantly higher mortality rate compared to early diagnosis, which is also evident in our study where all the patients who died presented after more than 24 h from symptom onset. However, in this study, it is unclear whether late diagnosis or advanced age was the primary prognostic factor, as the rather small sample size precluded a multivariable regression analysis. Moreover, older patients have received less aggressive treatment due to their age and preference. A recent meta-analysis combining results of iatrogenic and Boerhaave syndrome perforations showed that early treatment was associated with decreased overall mortality [26]. Sohda et al. also found a relationship between late diagnosis and leakage rates, noting that suture leakage is more likely in patients presented late or with a mediastinal abscess [10]. In our study, the differences in leakage or other complications between early and late diagnoses were not statistically significant. However, these findings may be attributed to the small sample size.

In the literature, primary surgical repair is still recommended as the optimal treatment approach regardless of the time of presentation [4, 10, 21, 27–30]. In our cohort study, primary surgical repair was deemed feasible in only one patient. Some studies have reported low leakage rates with primary repair [8]. Reinforced primary repair was also used to decrease the postoperative leakage rates [28]. However, the efficacy of reinforcement with flaps has contradictory outcomes in the literature and its value in delayed cases is also questioned [8, 10]. An alternative approach for delayed patients that showed promising results is repair with a T-tube to create a controlled esophago-cutaneous fistula [2, 28].

Although primary surgical repair is considered the cornerstone of treatment and should be the preferable approach, it is mostly not feasible due to delayed diagnosis and extensive inflammation and contamination in the area of the esophageal rupture [28]. Esophagectomy used to be the alternative treatment option in such cases. In our study, only one patient underwent esophagectomy as a last resort due to severe necrosis.

Contrary to previous reports, endoscopic treatment was used in patients diagnosed later than 24 h and as a salvage procedure in the most recent literature [13, 14, 21, 23]. In our cohort, urgent endoscopy was performed in all patients, irrespective of the time of diagnosis, to evaluate the feasibility of stenting for leak control. Esophageal leakage occurred in 4 out of 21 patients (19%), aligning with findings in the literature [14, 16, 27]. Also, these studies emphasize the importance of drainage as part of the treatment strategy of stenting, either surgical or image guided. Notably, Aloreidi et al. reported promising outcomes in patients with extensive mediastinal contamination through urgent drainage [14]. In our study, surgical drainage, when combined with stent placement, was required in 76% (16 out of 21 patients, of which 10 as primary treatment and 6 as additional treatment) of the cases. In fact, the majority of the patients in the stenting-only group (5 out of 8, 63%) eventually required surgical drainage, suggesting that surgical drainage should be an integral part of the treatment strategy.

Endoscopic vacuum therapy (EVT) has recently emerged as an alternative endoscopic technique that provides active drainage and secondary closure of the defect through negative pressure applied to an endoscopically placed sponge, either intracavitary or intraluminal. Its efficacy and safety have been demonstrated in several studies, yielding promising results, particularly in the treatment of anastomotic leakages [31–34]. However, its application in esophageal perforation, especially on Boerhaave syndrome, remains less well defined, with evidence primarily limited to recent studies and case series [35, 36]. The largest study to date using EVT as a primary treatment for Boerhaave syndrome involved 25 patients and reported a mortality rate of 8%. Notably, 14 of these patients required adjuvant surgical drainage, underscoring that EVT’s main advantage—effective sepsis control—often works best in conjunction with surgical drainage [35]. Despite its benefits, several concerns are associated with EVT. This techniques requires frequent sponge changes, posing a burden for patients and caregivers, and carries a risk of major hemorrhage, which can be life-threatening [37, 38]. Additionally, less severe but nonetheless significant adverse events such as sponge dislocation, minor bleeding, and the development of anastomotic strictures have been reported [39, 40]. These complication, while not fatal, can negatively affect patient quality of life and may require additional interventions.

While endoscopic treatment appears promising and can reduce the need for resection, surgery remains the most commonly used treatment modality for Boerhaave syndrome in the literature. Nonetheless, there is a growing trend toward the use of stents [16, 30]. A nationwide study conducted in the USA by Thornblade et al. reported an increase in the utilization of esophageal stents for benign esophageal perforations, rising from 7% in 2007 to 30% in 2014 [30]. However, no improved outcomes were observed despite the increasing use of stents [30]. Therefore, further research is required to establish clear criteria and optimize patient selection for stent use.

In an effort to guide the management of esophageal perforation, University of Pittsburgh developed the Pittsburg Perforation Score (PSS) initially based on expert opinion and later validated with clinical data. The score includes 10 risk factors, among which clinical signs of sepsis and extent of mediastinal/pleural contamination, but it is not specific to the etiology of the esophageal perforation [41]. Patients treated nonoperatively typically had a lower score than patients treated operatively. While a large multicenter study by Schweigert et al. demonstrated strong predictive accuracy for morbidity and mortality, another study found limited associations, with some factors (e.g., cancer) even inversely related to outcomes [20, 42]. Our analysis did not demonstrate a clear correlation between the PSS score and treatment outcomes in Boerhaave syndrome either. These inconsistencies highlight the need for prospective validation in larger cohorts to refine the PSS and better assess its utility as a decision-making tool. In addition, the difficulty in diagnosing Boerhaave syndrome limits this tool’s applicability in initial interventions.

**Fig. 3:**
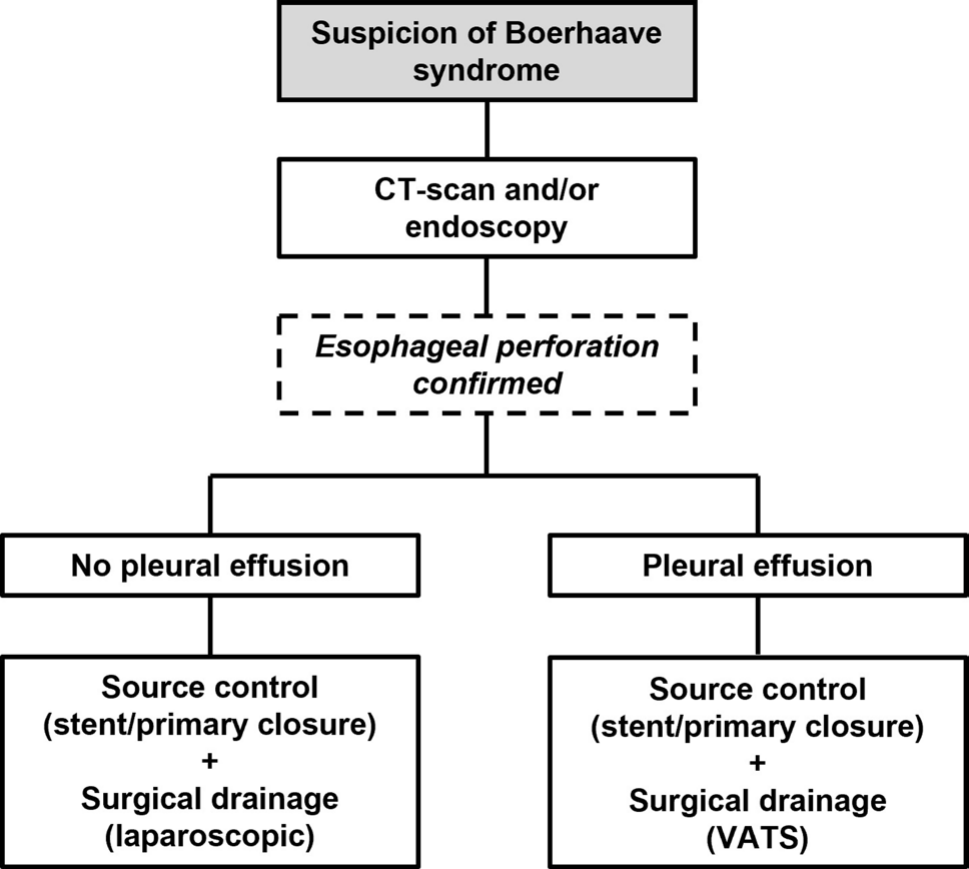
The proposed treatment algorithm for the management of Boerhaave syndrome

In the treatment of Boerhaave syndrome, preservation of organ function and decrease of mortality and morbidity should be the primary treatment objectives. The experience of our tertiary center in treating Boerhaave syndrome, as presented in this study, aimed to provide clinical guidance for its management, given the current lack of standardized guidelines. While the literature is not conclusive, it suggests that primary surgical repair, stenting, and EVT are effective closure strategies to seal the perforation and avoid resection surgery. In line with literature, our approach has shifted over time to prioritize minimally invasive techniques, as evidenced by the fact that we performed only one esophagectomy [43]. However, there is consensus that all those techniques should typically be accompanied by drainage, as also demonstrated in our study. Therefore, a multimodality treatment approach that includes a closure technique to initiate damage control and seal the perforation site, followed by surgical drainage should be recommended [24]. A combined procedure is preferred, also because an expanded stent can obstruct transhiatal drainage. This recommendation applies to both early- and late-diagnosed patients; however, the outcomes appeared to be notably worse in the late-diagnosed patients. Based on our results and the literature, we have developed a treatment algorithm, which is presented in Fig. 3.

This study has certain limitations, such as the relatively small number of patients identified due to the rarity of the syndrome and the heterogeneity in treatment strategies, which limits the analysis to a descriptive approach. In addition, information bias is introduced by the retrospective design and referral of included patients from other centers in the Netherlands. Selection bias is another limitation, as included patients were those presenting to or referred to our center, potentially limiting generalizability. Temporal bias also exists, with evolving management strategies during the study period, including increased use both minimally invasive techniques and changes in multidisciplinary approaches, such as antibiotic regimens, which could impact outcomes. Lastly, the lack of follow-up data restrict assessment of long-term outcomes. Despite these limitations, this study boasts significant strengths. Notably, we not only employed a systematic approach to identify patients treated within our center, minimizing the risk of overlooking cases, but we also ensured complete information by obtaining necessary data from referred patients to conduct this study comprehensively.

This study underscores the critical role of a multidisciplinary approach in managing Boerhaave syndrome. Based on our findings, we developed a treatment algorithm to guide clinical practice, recommending the prioritization of source control (defect closure) combined with surgical drainage, regardless of whether the diagnosis is early or late. Minimally invasive techniques should be employed whenever possible. Future multicenter studies are required to standardize treatment protocols, minimize bias, and provide robust data that can further guide clinical decision-making in the management of Boerhaave syndrome.
